# 

**Published:** 1914-04-18

**Authors:** 


Mr. John Gilbert Crompton, J.P., banker, formerly Mayor of Derby and High Sheriff of the county, has left £2,000 to Derbyshire Royal Infirmary and £500 to Wirksworth Cottage Hospital.

### Appointments.

Mr. A. E. Barnes, M.B., Ch.B. (Sheffield), M.B. (London), M.R.C.P., has been appointed to the post of Lecturer in Materia Medica, Pharmacology, and Therapeutics at the University of Sheffield.

### AN OFFER TO HOSPITAL LIBRARIES.

We are asked to state that a copy of "Letters from Norway," by Sir Walter Miéville, K.C.M.G., pp. 200, cloth, will be sent to hospitals in the United Kingdom on application being made to the Publishers, Combridges, Hove, Sussex, if 3d. be enclosed to cover postage.

### Gifts and Bequests.

Sir Riley Lord has secured the sum of over £500 in subscriptions to a fund for the endowment of a bed in the King Edward Ward of the Royal Victoria Infirmary, Newcastle-upon-Tyne. These contributions will enable the infirmary committee to claim the balance of £1,000 promised to complete the endowment of one bed. If the subscription reaches £1,000 or over the donor is willing to add the balance of another £1,000 for the purpose of endowing a second bed. The memorial is all the more appropriate since King Edward opened this infirmary accompanied by Queen Alexandra, and sanctioned the names of two of the wards in honour of the visit.

Mr. Richard Gunter, of West Hoathly, Sussex, has left £100 to the Royal Sussex County Hospital; also £100 and a silver salver with the "Gunter" arms to Surgeon-Major F. E. Gunter, R.A.M.C.

Mr. William Raven, hosiery manufacturer, of Leicester, has bequeathed £1,000 each to the Leicester Infirmary and the National Association for the Prevention of Consumption.

Mrs. Grace Catherine Pakenham Mahon, of Westbrook, near Ryde, Isle of Wight, and of Strokestown, Ireland, has left £3,000 to the British Home for Incurables at Streatham.

### The Book Market.

LIST OF NEW BOOKS RECEIVED FROM THE FOLLOWING PUBLISHERS:—

G. Bell and Sons: "Infant Care." By Kate Truelove.

J. and A. Churchill: "School Lighting." By Elwin H. T. Nash, D.P.H.

E. Marlborough and Co.: "German Technical Words and Phrases." By Thimm and Knoblauch.

J. Nisbet and Co.: "Surgery of the Stomach." By Herbert J. Paterson, M.A., M.C., F.R.C.S.

Sir I. Pitman and Sons: "Chart of the National Health Insurance Acts."

J. Wright and Sons: "Medical Annual, 1914."

### Institution and Public Reports.

A Report upon the Biology of Syphilis. By J. E. R. McDonagh, F.R.C.S. Printed by Harrison and Sons, 45-47 St. Martin's Lane, W.C.

First Annual Report on Tuberculosis in the County of Hertford for 1913. By H. Hyslop Thomas, County Tuberculosis Officer.

THE

### Doctors' Wives Association.

(*See page* 59.)

### COUPON.

I hereby certify that I am a doctor's wife, and I beg to send you my name and address as one who wishes to become a member of the proposed Doctors' Wives Association. I hereby agree to qualify as a member by paying the subscription of 7*s.* 6*d.* per annum on or before May 1, 1914, such subscription entitling me each year to a free copy of The Hospital newspaper delivered weekly, with the Doctors' Wives section, and to include my annual subscription to the Association.

This agreement undertaking to subscribe on my part is subject to at least 1,000 doctors' wives conditionally joining the proposed Association by signing and returning to "Co-operation" as below by April 18 next a similar coupon to that to which I hereby attach my signature.

(Signed) ..............................

(Wife of) * ..............................

(Address) ..............................

..............................

(Date) .................. ..................

*Note.*—When filled in and signed cut out this coupon, place it in an envelope and address and post it to:

Co-Operation,
c/o The Scientific Press,
28 & 29 Southampton Street, Strand,
London, W.C.

* Here insert full name and qualifications.

Rates of Subscription: Twelve months, 6 6; Six months, 4/-; Three months, 2/-, post free to any address in the United Kingdom. Abroad, Twelve months, 8/8; Six months, 5/-; Three months, 2/6, post free.—Address The Subscription Dept., "The Hospital," The Hospital Building, 28/29 Southampton Street, Strand, London, W.C.

